# Biologically Based Intelligent Multi-Objective Optimization for Automatically Deriving Explainable Rule Set for PV Panels Under Antarctic Climate Conditions

**DOI:** 10.3390/biomimetics10100646

**Published:** 2025-09-25

**Authors:** Erhan Arslan, Ebru Akpinar, Mehmet Das, Burcu Özsoy, Gungor Yildirim, Bilal Alatas

**Affiliations:** 1 Scientific and Technological Research Council of Türkiye, Marmara Research Center, Polar Research Institute, Gebze 41470, Türkiye; 2 Mechanical Engineering Department, Firat University, Elazig 23119, Türkiye; m.das@firat.edu.tr; 3 Maritime Faculty, Istanbul Technical University, İstanbul 34940, Türkiye; 4 Computer Engineering Department, Engineering Faculty, Firat University, Elazig 23279, Türkiye; 5 Software Engineering Department, Engineering Faculty, Firat University, Elazig 23279, Türkiye

**Keywords:** Antarctica Horseshoe Island, renewable energy, Turkish Antarctic Expedition, biologically based algorithm, photovoltaic, intelligent optimization

## Abstract

Antarctic research stations require reliable low-carbon power under extreme conditions. This study compiles a synchronized PV-meteorological time-series data set on Horseshoe Island (Antarctica) at 30 s, 1 min, and 5 min resolutions and compares four PV module types (monocrystalline, polycrystalline, flexible mono, and semitransparent) under controlled field operation. Model development adopts an interpretable, multi-objective framework: a modified SPEA-2 searches rule sets on the Pareto front that jointly optimize precision and recall, yielding transparent, physically plausible decision rules for operational use. For context, benchmark machine-learning models (e.g., kNN, SVM) are evaluated on the same splits. Performance is reported with precision, recall, and complementary metrics (F1, balanced accuracy, and MCC), emphasizing class-wise behavior and robustness. Results show that the proposed rule-based approach attains competitive predictive performance while retaining interpretability and stability across panel types and sampling intervals. Contributions are threefold: (i) a high-resolution field data set coupling PV output with solar radiation, temperature, wind, and humidity in polar conditions; (ii) a Pareto-front, explainable rule-extraction methodology tailored to small-power PV; and (iii) a comparative assessment against standard ML baselines using multiple, class-aware metrics. The resulting XAI models achieved 92.3% precision and 89.7% recall. The findings inform the design and operation of PV systems for harsh, high-latitude environments.

## 1. Introduction

Antarctica, despite its extreme environmental conditions, offers a unique setting for scientific research and environmental monitoring, particularly in understanding global climate dynamics and as a natural laboratory of the World. The region serves as a critical observatory for assessing the impacts of climate change in polar environments and for modeling future global climate scenarios. However, the energy infrastructure that supports scientific operations in Antarctica remains largely dependent on fossil fuels. This reliance not only presents a contradiction in terms of environmental sustainability but also introduces significant logistical challenges. Transitioning to renewable energy sources is, therefore, not merely a matter of reducing carbon emissions, but also a strategic imperative for enhancing operational efficiency and autonomy in remote research settings [1,2].

There is a growing interest in the implementation of renewable energy sources to meet energy demands in Antarctica. During the austral summer, the region benefits from extended daylight periods and predominantly clear skies, which result in low but continuous solar radiation [3]. The utilization of this solar energy through photovoltaic (PV) panels has the potential to significantly reduce both carbon emissions and logistical costs. In their study on wind energy potential, Bolonkin and Cathcart highlighted that certain regions of Antarctica possess favorable conditions for wind power generation, suggesting that wind energy could make a meaningful contribution to renewable energy systems in the region [4]. Moreover, hybrid energy systems represent a promising solution for achieving energy autonomy under Antarctic conditions. Babinec et al. demonstrated that integrating solar and wind power could reduce the reliance on diesel generators by up to 90% [5].

Since 2017, Turkey has increasingly strengthened its scientific presence in Antarctica through a series of national expeditions. During these missions, the establishment of the “Turkish Science Camp” has served not only as a research base but also as a platform that demonstrates the feasibility of conducting sustainable scientific operations under the harsh conditions of Antarctica [6]. Following the camp’s establishment, an Environmental Impact Assessment (EIA) Report, emphasizing environmental sensitivity, was submitted to the international community and remained valid for a period of three years [7]. This initiative reflected Turkey’s responsible and constructive approach to Antarctic research. In parallel, an automatic weather observation system (AWOS) was installed on Horseshoe Island in cooperation with the Turkish State Meteorological Service, adding a new dimension to ongoing scientific efforts [7]. Powered by renewable energy, the AWOS supports environmental sustainability goals and reliably collects meteorological data under extreme Antarctic conditions. The data generated by the AWOS include key parameters such as wind speed and direction, air temperature, relative humidity, actual pressure, solar radiation, and sunshine duration. In a region where maximum wind speeds of up to 327 km/h have been recorded, the effective operation of such a system highlights the robustness of Turkey’s engineering and scientific infrastructure [8].

**Figure 1:**
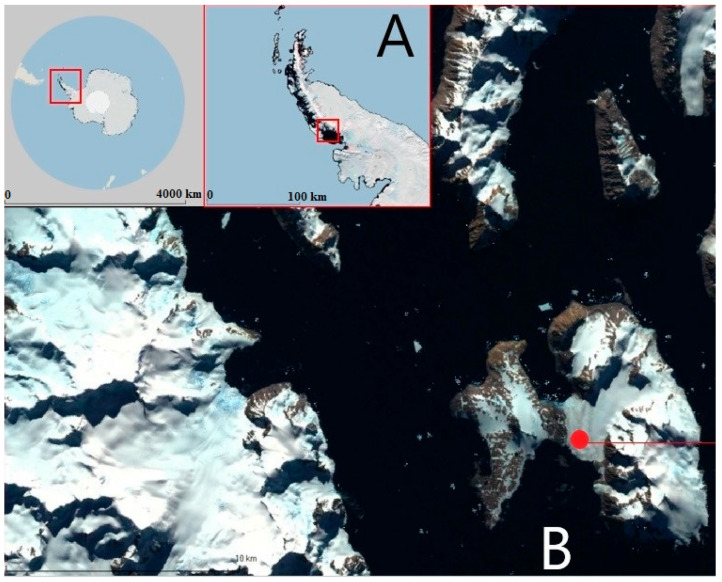
Logistical route of the Turkish Antarctic Expedition (TAE) (**A**), and Turkish Antarctic Research Station (TARS) (**B**).

Figure 1A shows the logistical route of the Turkish Antarctic Expedition, while Figure 1B indicates the location of Horseshoe Island in the region and the location of the Turkish Antarctic Research Station (marked with a red dot). These images highlight the strategic geographical location in which Turkish scientists are working. In conclusion, the work carried out by the Turkish Antarctic Exploration Team not only demonstrates Turkey’s scientific capabilities but also highlights its contribution to global research. These activities prove that a sustainable scientific approach is possible in harsh environmental conditions and that innovative solutions can lead to greater success. The experiments conducted within the scope of this study took place on Horseshoe Island, located in Marguerite Bay on the Antarctic Peninsula. The geographic coordinates of the study site are 67.829676° South latitude and 67.237757° West longitude (coordinates: 67°49′46.83″ S, 67°14′15.92″ W). This location hosts the Turkish Science Camp, which serves as a temporary research station. The experiments were carried out during the Antarctic summer season—typically encompassing December, January, and February—specifically in February 2024. Figure 1 provides an overview of the area surrounding the Turkish Antarctic Research Station (TARS). In the figure, the red box outlines the Antarctic Peninsula, the circular marker denotes Marguerite Bay, and the red dot indicates the exact location of the experimental site.

### 1.1. Utilization of Renewable Energy for Antarctic Research Stations

The integration of renewable energy in Antarctic research stations is of paramount importance, not only for environmental sustainability but also for ensuring energy independence. Traditionally, these stations have relied on fossil fuels such as polar diesel, which entail high operational costs and significant logistical challenges [9]. The adoption of renewable energy technologies enables the utilization of cleaner, more reliable, and environmentally friendly energy sources, even under the extreme conditions of Antarctica.

For example, a study conducted at Neumayer Station III proposed a hybrid system combining solar and wind energy, demonstrating a significant potential reduction in annual CO_2_ emissions [9]. Similarly, research carried out for China’s Zhongshan Station designed a standalone renewable energy system, highlighting the long-term operational reliability such systems can offer [10]. At Concordia Station, a hybrid energy system that integrates solar and wind sources was shown to enhance energy security on glacial surfaces under extreme conditions [2]. Moreover, a comprehensive study that mapped the energy profiles of all research stations in Antarctica and assessed the adoption rate of renewable energy systems concluded that although the use of such technologies is on the rise, they still require broader institutional and infrastructural support [11]. At Esperanza Base, an investigation into vertically oriented solar panels facing northeast (NE) and northwest (NW) revealed that vertical alignment reduces snow accumulation and mitigates seasonal energy production fluctuations. A PV system supported by a hydrogen storage unit was able to meet 94% of the station’s annual energy demand, reducing fossil fuel consumption by 1000 L [12]. In another study, an optimized distributed energy system composed of solar panels, wind turbines, and diesel generators achieved an 11.8% reduction in total energy consumption [13]. Research at SANAE IV Station examined the technical and economic feasibility of PV systems composed of 143 m^2^ of flat-plate solar collectors with a capacity of 40 kWp. While the findings emphasized the long-term energy independence potential of PV systems, they also revealed that solar thermal systems offer greater economic advantages [14]. A further investigation into the combined use of PV panels and wind turbines under Antarctic conditions found that wind turbines provide a continuous energy source throughout the winter months, filling the gap in solar energy availability [1]. On Horseshoe Island, a study analyzing the energy and exergy efficiency of a standalone PV panel found its energy efficiency ranged between 5.40% and 11.35%, contributing meaningfully to the reduction of CO_2_ emissions [15]. Additionally, a study conducted in the Qaanaaq region evaluated the seasonal sensitivity of rooftop PV panels, demonstrating that they could meet up to 57% of the region’s annual energy demand [16]. Yakutia, a field study, analyzed the impact of snow accumulation on polished and unpolished PV panels, revealing its considerable influence on energy output [17].

In conclusion, renewable energy solutions have significant potential in meeting the energy needs of Antarctic research stations and reducing their environmental impacts. These solutions not only alleviate logistical challenges but also significantly reduce the carbon footprint. Efforts in Antarctica create a model for global studies on renewable energy applications in extreme environmental conditions.

### 1.2. PV Power/Efficiency Modeling: Physical Approaches and Data-Driven Methods

The utilization of renewable energy in challenging geographies such as Antarctica not only ensures environmental sustainability but also enhances the reliability of logistical operations. When combined with advanced technologies such as artificial intelligence (AI) and machine learning, these efforts have the potential to revolutionize energy efficiency and system resilience. AI-supported systems are being used to optimize the variability of renewable energy sources and to make energy management more flexible.

Arslan et al. developed a model using the M5P algorithm to enhance the energy efficiency of PV panels in Antarctica. The M5P referred to in this study is the M5′ (M5-prime) version of Quinlan’s M5 model trees, developed by Wang and Witten [18]. This model has provided a more sustainable energy solution by optimizing the energy, exergy, and environmental-economic analyses of the system [15]. Lösing and Ebbing improved the infrastructure of renewable energy projects in Antarctica by predicting geothermal heat flow using machine learning models [19]. Polatoğlu correlated cloud cover predictions with solar energy production using LSTM models [20]. The aim of his study is to improve solar energy forecasts in regions with high cloud cover, such as Antarctica [21]. Zeng et al. conducted a long-term analysis of surface solar radiation at Zhongshan Station, using artificial intelligence and data analysis methods to estimate the solar energy production potential in Antarctica [22]. Hakimi et al. developed an artificial intelligence-based method for short-term surface wind speed prediction in different climate regions using ERA5-based datasets. In the study, they demonstrated the model’s performance in different geographies using neural networks and presented that high accuracy (R^2^ ≈ 0.99) was achieved even in extreme conditions such as Antarctica. Although the Antarctic datasets (2022 and 2024) contained wide wind speed ranges and high variability, the model demonstrated both interpolation and extrapolation capabilities. They also emphasized the need to increase transparency in the future with interpretable artificial intelligence techniques [23]. Sidhaarth et al. report significant accuracy improvements in short-term wind speed prediction by combining multivariate deep learning models with enhanced MissForest imputation; it provides a methodological reference for managing time series gaps frequently encountered at polar/remote stations due to sensor outages and rapid meteorological variability [24].

This work is driven by the goal of building an AI framework that is both highly accurate and interpretable for estimating the solar energy potential achievable under Antarctic conditions. While opaque (“black-box”) models often deliver strong forecasts, they seldom clarify how outputs arise or which inputs matter, limiting transparency and confidence in engineering decisions for renewable systems. Here, we seek interpretable, data-driven rules derived from experiments on a testbed comprising monocrystalline, polycrystalline, flexible monocrystalline, and transparent PV modules, all with identical aperture areas, to characterize PV potential in Antarctica. The results indicate that, even in severe climates, achieving energy self-sufficiency is feasible and that optimized energy management for regional research stations is within reach.

The study contributes in two principal ways. First, it offers a head-to-head comparison of four PV technologies under Antarctica’s distinctive environment, addressing a notable gap in prior work on renewable feasibility in extreme settings. Second, it boosts the accuracy and reliability of PV performance predictions using advanced machine-learning models; in particular, rule sets automatically discovered by a modified multi-objective SPEA-2 procedure perform robustly under challenging conditions. Collectively, the findings demonstrate the promise of explainable AI for optimizing PV operation and advancing sustainable power solutions, thereby improving renewable energy projections and providing trustworthy guidance for deployment in harsh environments.

## 2. Materials and Methods

### 2.1. Experimental Setup

**Figure 2:**
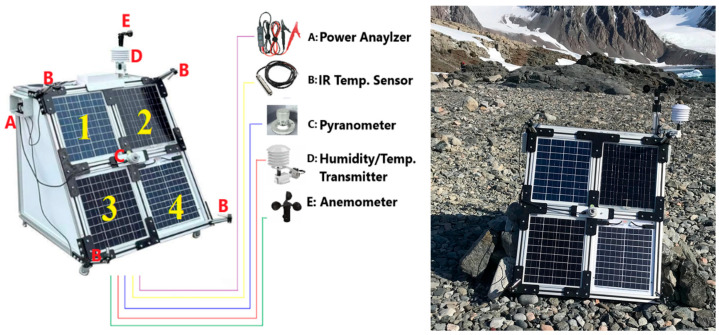
Sensor specifications and experimental setup (1-Polycrystalline, 2-Flexible Monocrystalline, 3-Monocrystalline, 4-Semitransparent).

Field experiments were carried out on Horseshoe Island during the austral summer months (December–February). The instrumentation and layout adjacent to TARS are illustrated in Figure 2. Because hardware longevity is critical in the harsh Antarctic environment, the system employed two batteries wired in parallel. A resistive dummy load was used to draw current from the PV array; three resistors, connected in series (10 Ω, 10 Ω, and 3.3 Ω), provided rapid electrical loading. To minimize ambient influences (air temperature, humidity, etc.), these loads were placed inside a thermally insulated, secondary enclosure that approximated adiabatic conditions.

A suite of sensors was integrated to capture key meteorological and system variables. A solar radiation sensor was mounted at the center of the rig (marker C). Wind direction/speed instrumentation (marker E) and a combined temperature–humidity probe (marker D) were installed on the upper section. Each PV module was equipped with a forward-facing infrared thermometer to monitor the module surface temperature (marker B), utilizing a total of four IR sensors. After installation, temperatures measured at the metallic interconnects were compared against panel-surface readings to verify accuracy. An LCD enabled live observation, while a data logger continuously recorded meteorological parameters, module surface temperature, and electrical quantities—voltage (V), current (I), and power (W)—in real time (marker A).

Performance (efficiency) measurements were obtained for four PV technologies—polycrystalline (1), flexible monocrystalline (2), monocrystalline (3), and semitransparent (4)—each configured for reliable operation under Antarctic conditions.

Conducting scientific studies in extremely harsh climatic conditions such as Antarctica necessitates a meticulous approach in the selection of the equipment used. In this context, the PV test system equipment listed in Table 1 has been carefully selected to withstand low temperatures, high wind speeds, and harsh environmental conditions. All equipment has a wide operating temperature range of −20 °C to +80 °C. This broad range ensures the reliable operation of systems under Antarctica’s variable weather conditions.

The sensitivity values of the sensors listed in Table 1 demonstrate the careful consideration that went into this selection. In choosing the sensors, not only durability but also the accuracy of the measurements were critical criteria. Sensors capable of collecting data with the lowest margin of error, even under challenging environmental conditions, were preferred. This approach ensures that the scientific studies conducted in Antarctica maintain high-quality standards.

The data presented in Table 2 detail the technical specifications of four different PV panels capable of generating electricity from the sun. Table 2 lists the manufacturer/catalog-based technical specifications of the four PV modules used, not the measurement results obtained from the experiments. The 25 W value here is a nominal value representing the installed/nameplate power of all modules and is valid under standard test conditions (1000 W/m^2^, AM 1.5, cell temperature 25 °C); this does not mean that the modules always produce 25 W. In field tests, instantaneous power continuously varies depending on environmental conditions such as global solar radiation, cell temperature, wind, and angle; the dataset includes these measured time series. To enable a fair technology comparison, all modules were selected from the same nominal power class (25 W). These panels have a surface area of 0.12 m^2^ and are fixed onto the same platform. Having all panels on the same surface area and common platform allows for testing the different panel types under equal conditions, facilitating the attainment of comparable results. When determining the tilt angle of the panels, the Hottel & Woertz (HW) method was employed, and this angle was optimized to 54.3° in the northwest direction [25]. This method aims to maximize annual solar radiation in order to enhance panel efficiency, thereby optimizing the energy production potential of the panels even under the challenging conditions of Antarctica. The adherence of the panels to the platform at this angle demonstrates a meticulous engineering approach in the design of the system.

### 2.2. Uncertainty Analysis

Measurement quality comprises two distinct aspects: accuracy, the closeness of a reading to the actual quantity, and precision, the extent to which repeated observations under the same conditions agree with one another. A thorough accounting of all influences on measured outcomes supports sound choices in experimental design and in selecting the instruments to be used. To quantify these influences, we applied an uncertainty evaluation to the experimental dataset, following the propagation procedure introduced by Kline and McClintock [26,27].

In this study, an uncertainty budget was created for electrical quantities related to PV panels. Module efficiency was calculated as the ratio of the product of the voltage (V) and current (I) measured from the panel to the product of the incident solar radiation (UV) and the total PV area (A_pv_), as expressed in Equation (1). When evaluating efficiency uncertainty, the sensor-induced error term (Es) arising from communication between sensors and the data logger was also included in the calculations. The total uncertainty results from current, voltage, PV surface area, radiation, and sensor contributions are presented in Table 3 for monocrystalline (MPV), polycrystalline (PPV), flexible (FPV), and transparent (TPV) module types.

### 2.3. Calculation Procedure

In order to enhance our understanding of the performance of flat-plate photovoltaic (PV) modules, we conducted a systematic measurement of temperature, voltage, and current through carefully designed experimental procedures. From these records, both energy and exergy efficiencies were calculated, and the resulting efficiency values were used to conduct an environmental assessment.

To characterize the performance of the flat-plate PV module, we recorded temperature, voltage, and current during testing. These measurements were then used to determine the system’s energy efficiencies. Energy efficiency for PV systems is quantified as the ratio of useful electrical output to the solar energy incident on the module surface. In accordance with the first law of thermodynamics, the calculation uses *UV* and *A_PV_* (Equation (1)) [25].

(1) $$\eta_{\mathrm{PV}\;\mathrm{energy}}=\frac{P_{e}}{Q_{s}}=\frac{V\times I}{\mathrm{UV}\times A_{\mathrm{PV}}}$$

The measured electrical power output *P_e_* is the result of the product of the voltage and current produced by the PV systems. However, the measured voltage *V* and current value of the instantaneous PV panels are expressed by *I*. The electrical exergy of the PV system aims to use the available energy as useful energy.

### 2.4. Data Set and AI Procedure

This study involved estimating solar panel power using data collected from experiments performed on Horseshoe Island in Antarctica during the 8th National Antarctic Scientific Expedition. The dataset comprises temporal data, environmental variables (temperature, humidity, solar radiation, and wind speed), and power output values for four distinct panel types. The dataset was compiled only from data collected on sunny days throughout the Antarctic summer season. Inclement weather conditions hindered the execution of experiments throughout the voyage. Experiments performed under predominantly overcast conditions from 14 to 18 February demonstrated that PV panels were capable of generating electrical power. All data were utilized in the artificial intelligence process to evaluate the energy production performance of PV panels in the study results.

In this study, the data was used in its raw form because it was automatically recorded, time-stamped, and complete from a programmable logic control (PLC)-based infrastructure; no pre-processing steps, such as data cleaning, missing value imputation, resampling, filtering, or outlier removal, were applied. Only integrity checks that do not involve transformation (unit and tag matching, time synchronization with NTP, alarm/limit violation checks) were performed; thus, raw data integrity and traceability were preserved to prevent potential bias.

The dataset provides an alternative option to analyze the sensitivity of PV systems to environmental conditions. The study is confined to periods of solar radiation availability, as data were collected exclusively during daytime hours. The experimental results derive from 2380 observations, encompassing 20 parameters that include fundamental meteorological factors such as temperature, humidity, wind speed, and solar radiation, alongside surface temperature and instantaneous power output of four distinct panel types, recorded.

#### 2.4.1. Dependent and Independent Variables

The responses of interest are the panel power outputs (in watts) defined per module class. In the code, these are enumerated in the target columns dictionary as Poly PV, Mono PV, Transparent PV, and Flexible PV. Output power serves as the primary indicator of how each technology performs under changing environmental conditions.

Predictor variables—treated as independent inputs—are listed in feature columns and capture key environmental drivers: ambient temperature (TEMP), relative humidity (HUM), solar radiation (UV), and wind speed (SPEED). These external factors shape both the electrical yield and the thermal state of the modules. Using these predictors, the model learns rule-based relationships to estimate the power capability of each PV type across a range of meteorological and atmospheric scenarios.

#### 2.4.2. Random Rule Search and Modified SPEA-2 Algorithm for Multi-Objective Optimization

Multi-objective optimization (MOO) strategies address solutions that seek to simultaneously optimize many competing objectives [20]. The fundamental premise of these methods is to identify a solution set generated by the trade-off among various intended objectives. The quantity of opposing objectives enables the pertinent challenge to be categorized as multi-faceted or numerous. Despite the existence of several taxonomies, these methods can typically be assessed under three overarching categories: Priori, Posteriori, and hybrid methods. In Priori approaches, decision-makers participate in the solution process from the outset. Decision-makers can prioritize objectives or consolidate numerous objectives into a singular objective through the application of specific ratios. Conversely, the involvement of decision-makers in these methodologies introduces issues of subjectivity and constrained investigation. In posteriori approaches, the objectives are regarded with equal importance, and a solution set is provided that encompasses non-dominated outcomes. The decision-maker assesses and determines by analyzing this set of solutions. These methodologies are appropriate for scenarios in which the decision maker lacks information regarding the objectives or when the desired objectives hold equal significance. Given the absence of prior knowledge regarding the data set in data mining challenges, it is more suitable to employ posterior methods for random search. The predominant mechanism in posteriori approaches is the formation of the Pareto front [28]. The non-dominance criterion among candidates is crucial for establishing the Pareto front. In a maximizing problem like the one presented in this study, the dominance criterion is articulated as shown in Equation (2). If this requirement is satisfied, the solution $\vec{s_{1}}$ supersedes $\vec{s_{2}}$. In this context, *k* denotes the number of objectives, *i* signifies the pertinent objective index within the solution vector, and *f(.)* indicates the fitness function that determines the fitness value for the candidate.

(2) $$\forall i\in \left\{1,2,3,\ldots ,k\right\},\;\left[F\left(s_{1}^{i}\right)\geq f\left(s_{2}^{i}\right)\right]\cap [\exists i\in 1,2,3,\ldots ,k:f\left(s_{1}^{i}\right)>f\left(s_{2}^{i}\right)]$$

The method of trade-off evaluation of the Pareto curve obtained in the random search process is performed with MOO algorithms. This study preferred the Strength Pareto Evolutionary Algorithm 2 (SPEA2) algorithm, which has proven its strength in this field, and adapted it to rule mining [29]. SPEA2 offers faster convergence than classical SPEA with the elitism and tournament mechanisms it uses, while also enabling the formation of a more successful Pareto front with an advanced density estimation method.

SPEA-2 begins at generation t = 0 with an initial population ($P_{0}$) and an empty external archive (${\bar{P}}_{0}$). Both sets are adaptive: at each iteration, they are updated to ($P_{t}$ and ${\bar{P}}_{t}$) based on Pareto dominance tests and the algorithm’s update rules. Selection across generations is driven by fitness values assigned to individuals in both the population and the archive. When multiple candidates are dominated by the same archive members and exhibit similar fitness, the search becomes overly crowded; to counter this, SPEA-2 accounts for both the dominating and dominated relations of every candidate. The strength of the iii-th individual is defined as the number of solutions it dominates within $P_{t}$ and ${\bar{P}}_{t}$, and is computed by Equation (3). In this context, $\left|.\right|$ denotes set cardinality, “+” indicates the union/aggregation of sets, and “≻” denotes Pareto dominance.

(3) $$S\left(i\right)=\left|\left\{\left.j\right|j\in P_{t}+{\bar{P}}_{t}⋀i≻j\right\}\right|$$

$S\left(i\right)$ value is used to calculate the raw fitness $R\left(i\right)$ value that explains the dominance status of the i-th individual. The higher the $R\left(i\right)$ value found with Equation (4), the more likely the candidate is to be dominated. In the case of $R\left(i\right)$ = 0, the candidate is not dominated.

(4) $$R\left(i\right)=\sum_{j\in P_{t}+\bar{P_{t}},j≻i}S(j)$$

Density estimates also guide the interpretation of the raw fitness $R\left(i\right)$. To quantify density, a k-nearest-neighbor scheme is used. For each individual ith, we compute in the objective space the distances from iii to all other individuals j drawn from both the archive and the population, and collect these distances in a list. After sorting the list in ascending order, the k-th entry gives the neighborhood radius, denoted $\sigma_{i}^{k}$. The parameter k depends on the sizes of the population (N) and archive ($\bar{N}$) and is set to $k=\sqrt{N+\bar{N}}$. A candidate’s density term is inversely proportional to $\sigma_{i}^{k}$ and is evaluated via Equation (5), where θ  is a constant θ=2  used to keep the denominator positive. The final fitness is then obtained from Equation (6) by combining the raw fitness with this density contribution.

(5) $$D\left(i\right)=\frac{1}{\sigma_{i}^{k}+\theta }$$

(6) $$F\left(i\right)=R\left(i\right)+D(i)$$

The formation of the subsequent generation (environmental selection) is based on the $F\left(i\right)$ values of the candidates. Initially, as indicated in Equation (7), non-dominated individuals with a fitness value below 1 are transferred to the archive of the subsequent generation. The archive size of the subsequent generation is considered in this process. If ${\bar{P}}_{t+1}=\bar{N}$, the environmental selection process is concluded. If ${\bar{P}}_{t+1}<\;\bar{N}$, the optimal $\bar{N}-\;\left|{\bar{P}}_{t+1}\right|$ non-dominated individuals from the preceding archive and population are incorporated into the archive of the subsequent generation. If ${\bar{P}}_{t+1}>\;\bar{N}$ exceeds $\bar{N}$, candidates will be eliminated from the next generation’s archive until ${\bar{P}}_{t+1}=\bar{N}$.

(7) $${\bar{P}}_{t+1}=\left\{i\left|i\right.\in P_{t}+\bar{P_{t}}⋀F(i)<1\right\}$$

The candidate extraction method considers the distance ($\sigma_{i}^{k}$) of i in ${\bar{P}}_{t+1}$ to its k-th nearest neighbor, as illustrated in Equation (8), and identifies the individual with the minimal distance. If multiple candidate solutions exhibit identical distances, the distinction is made by evaluating the second smallest distances. Conversely, Simulated Binary Crossover (SBX) and fundamental mutation mechanisms are favored as the crossover operator in the algorithm [29].

(8) $$i\leq dj\Leftrightarrow \forall 0<k<\left|{\bar{P}}_{t+1}\right|:\sigma_{i}^{k}=\sigma_{j}^{k}\vee \exists 0<k<\left|{\bar{P}}_{t+1}\right|:\left[\left(\forall 0<l<k:\sigma_{i}^{l}=\sigma_{j}^{l}\right)⋀\sigma_{i}^{k}<\sigma_{j}^{k}\right]$$

The classical SPEA-2 algorithm cannot be directly applied in the field of data mining. Therefore, a well-designed representation scheme is required. In the modified SPEA-2, a candidate solution uses a representation composed of three different vectors. The candidate solution *V_i_* includes the sub-vectors ${\vec{V}}_{i}^{b},\;{\vec{V}}_{i}^{l}\;and\;{\vec{V}}_{i}^{u}$ (i= 1, 2, …, *n*). $V_{i}^{b}=[v_{1}^{b},v_{2}^{b},\;\ldots ,v_{j}^{b},\ldots ,v_{n}^{b}]$ is a binary vector that indicates which attributes will be used in the rule derived by the candidate. If the value of $v_{j}^{b}$ is greater than the predefined threshold “*λ*”, as shown in Equation (9), then the j-th attribute is included in the rule to be derived by the candidate.

(9) $$v_{j}=\left\{\begin{array}{c} 1\;\left(j\;nth\;quality\;rule\;exists\right)\;if\;v_{i}^{b}>\lambda ,\;\;\lambda \;\epsilon \;\left[0,1\right]\;\;\;\;\;\;\;\;\;\\ \;\;0\;(none)\;\;\;\;\;\;\;\;\;\;\;\;\;\;\;\;\;\;\;\;\;\;\;\;\;\;\;\;\;\;\;\;\;\;other\;\;\;\;\;\;\;\;\;\;\;\;\; \end{array}\right.$$

Throughout iterations, candidates obtain two values for each attribute of the search space, one lower and one upper. A candidate stores the lower/upper values found for each attribute in $V_{i}^{l}=\left[v_{1}^{l},v_{2}^{l},\ldots v_{j}^{l},\ldots v_{n}^{l}\right]\;and\;V_{i}^{u}=[v_{1}^{u},v_{2}^{u},\ldots v_{j}^{u},\ldots v_{n}^{u}]$ (${v_{j}^{l},\;v}_{j}^{u}\;\epsilon R$). In an iteration, the lower and upper values found for the j*th* attribute must remain between the minimum $\left(L^{l}\right)$ and maximum ${(U}^{u})\;$ values in the search space for the relevant attribute.

Therefore, the condition ${L_{j}^{l}\leq v}_{j}^{l}<\;v_{j}^{u}\leq U_{j}^{u}$ must be continuously checked. Candidates have a rule in each iteration, and the rules are updated throughout the iterations. The value of $v_{j}^{b}$ is decisive in rule formation, and $v_{j}^{l}$ and $v_{j}^{u}$ enable the rule to be interpreted. For example, in a search for an “S” class, let us assume that the 2nd, 4th, and 8th attributes are included in the rule in the *i*-th iteration ${\;(v}_{4}^{b},\;v_{7}^{b},\;and{\;v}_{10}^{b}>\;\lambda ).$ In this case, the candidate rule expression for the data set attributes $F_{1},\;F_{2},\;\ldots \;,\;F_{n}$ will be as shown in Equation (10).

(10) $$if\;v_{2}^{l}\leq \;F_{2}\leq \;v_{2}^{u}\;and\;v_{4}^{l}\leq \;F_{4}\leq \;v_{4}^{u}\;and\;v_{8}^{l}\leq \;F_{8}\leq \;v_{8}^{u}\;then\;S$$

The candidate’s performance evaluates the consistency of the rule it has derived for each data class. During the rule assessment, the “if” and “then” components of the rule derived from the *V_i_* candidate solution are considered. The attribute values and class of the compared data determine which segment or segments of the extracted rule are consistent. Following the comparison presented in Table 4, the metrics for true positive (TP), true negative (TN), false positive (FP), and false negative (FN) of the proposed solution are revised. These metrics are utilized in the computation of the candidate’s objective values. In multi-objective optimization techniques, the conflict among the chosen objectives is significant. The trade-offs arising from this paradox facilitate the establishment of the Pareto front. In data mining, Precision (Pre) and Recall (Rec) are metrics that may be in conflict with one another, and these two metrics, as presented in Equations (11) and (12), were considered in this work. In every iteration, the *i*-th candidate solution retains these two metrics $\vec{Os}=[Pre,Rec]$ within the objective vector. The dominance relationship among the candidates is established by comparing these objective vectors. Algorithm 1 presents the pseudocode illustrating all operations of the rule inference technique with SPEA2.

(11) $$Precision(Pre)=\frac{TP}{TP+FP}$$

(12) $$Recall(Rec)=\frac{TP}{TP+FN}$$

**Algorithm 1. Modified SPEA2 pseudocode**1.*Define all fixed and predefined parameters;*$\;N,\bar{N}$***,*** γ, φ, ∂***,***  λ2.***Initialize*** 
$P_{t}\;ve\;{\bar{P_{t}}}^{\prime }\;and\;for\;t=0,$ 
*create* 
$V_{i}(and\;{\vec{V}}_{i}^{b},{\vec{V}}_{i}^{l},\;\;{\vec{V}}_{i}^{u})$
3.*Create rules for each individual (Equation (9))*4.***While*** 
*(t < Maximum number of iterations)*
5.*Calculate the eligibility of individuals based on rule assessments (Equations (11) and (12) and Table 4)*6.*Transfer all non-dominating solutions (create*$\;{\bar{P}}_{t+1}$*)*7.***If*** $\;{\bar{P}}_{t+1}\;$*exceeds*$\;\bar{N}\;$***then*** $reduce\;{\bar{P}}_{t+1}\;$*(Equation (7))*8.***Else if*** ${\bar{P}}_{t+1}\;$***size less than***$\;\bar{N}\;$***then full*** ${\bar{P}}_{t+1}\;$*by non-dominated solutions*9.*SBX and mutation operations*10.*Update all individuals*11.*t* = *t* + 112.***End While***

The explainable multi-objective optimization framework is environment-independent since it is defined through environmental inputs. Transfer to conditions such as space or high-temperature regimes is possible through the rescaling of input variables and the redefinition of objective/constraint functions according to the relevant physical boundaries. Therefore, without any specific application, the formalism is scientifically compatible with data obtained from such environments.

Model selection prioritized algorithms capable of representing both linear trends and nonlinear interactions that govern the linkage between environmental drivers and PV module behavior. We organized the candidates into two families: regression-oriented methods—Elastic Net (EN) and Polynomial Regression (PR)—and an interpretable AI track comprising a Multi-Objective Optimization–Based Random Rule Search paired with a Modified SPEA-2 procedure.

While these approaches can deliver strong predictive performance, they also come with limitations. The dataset spans a relatively narrow time window collected under specific weather regimes, which restricts how confidently the models can be generalized to broader or different periods. In highly variable settings such as Antarctica—where conditions shift quickly and markedly—a longer observation campaign would have likely improved model robustness. By training each model with tuned hyper parameters and benchmarking their accuracy under varied conditions, this study provides a rigorous comparison of predictive capability and advances a more complete picture of how environmental factors influence PV panel performance.

## 3. Results

Scientific studies conducted under Antarctica’s extreme climate conditions clearly demonstrate the critical role of weather and environmental factors on experimental results. In this context, the unfavorable weather conditions on the days the experiments were conducted significantly limited the continuity of experiments focused on solar energy. Antarctica’s variable nature makes it difficult to carry out studies such as energy production and environmental analysis, but researchers’ ability to overcome these challenges adds significant value to the scientific process.

**Figure 3:**
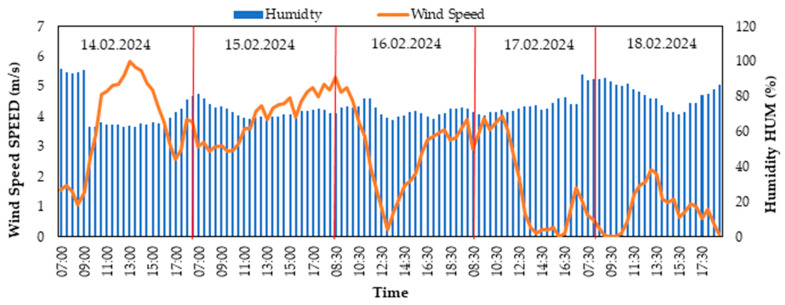
Antarctica Horseshoe Island wind-velocity and humidity values (14–18 February 2024).

Figure 3 shows that wind speed ranged from 0.4 to 5.8 m/s and relative humidity ranged from 70.7% to 95.3% during the period from 14 to 18 February 2024; this window indicates that mixing/advection processes and near-saturated humidity regimes dominated in the boundary layer. Epidemic increases in wind speed can trigger short-scale humidity fluctuations by accelerating the mixing of near-surface air masses; this can be observed in time series as sudden humidity drops (drying out mixing) or rises (humid air advection). High relative humidity levels narrow the difference between the dew point and ambient temperature; this is consistent with the likelihood of fog/condensation and low cloud base conditions.

**Figure 4:**
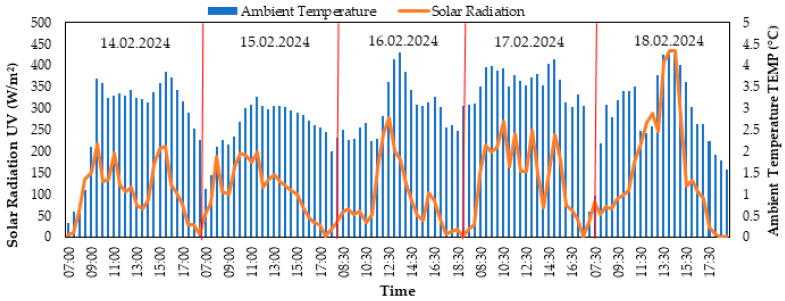
Antarctica Horseshoe Island solar radiation and air temperature changes (14–18 February 2024).

As shown in Figure 4, shortwave solar radiation varies between 17 and 442 W/m^2^, exhibiting a distinct day–night and cloudiness cycle; ambient temperature progresses with a limited thermal amplitude in the 0.5–4.4 °C band. A short-scale phase difference related to thermal inertia is expected between radiation peaks and troughs and the temperature response; the increase in radiation during clear intervals and the decrease during cloudy/humid intervals are consistent with this pattern. The narrow amplitude of temperature points to the possibility of heat exchange damped by strong mixing or marine effects in the lower troposphere; taken together, the two figures describe a meteorological regime dominated by short-scale cloud–advection dynamics.

When Figure 3 and Figure 4 are evaluated together, the fluctuations in shortwave solar radiation in the range of 17–442 W/m^2^ are the primary indicator of cloudiness and air mass changes, generally exhibiting an inverse relationship with relative humidity and a short delay-dependent positive relationship with ambient temperature due to thermal inertia. High relative humidity in the range of 70.7–95.3% coincides with radiation suppression during closed/humid episodes; conversely, a decrease in relative humidity is expected during open intervals, accompanied by increased radiation. A temperature remaining within a narrow band of 0.5–4.4 °C indicates the damping of daily thermal amplitude due to high humidity and surface-level mixing; the temperature response accompanies radiation peaks/troughs with a short phase difference. Increases in wind speed in the range of 0.4–5.8 m/s strengthen boundary layer mixing, triggering two possible patterns: (i) a short-term decrease in relative humidity with the transport of drier air and a temporary recovery in radiation with cloud breaks, (ii) an increase in relative humidity due to moist air advection and a weakening of radiation. Consequently, the diurnal climate signal is characterized by a regime dominated by cloudiness/advection dynamics, jointly modulated by the “radiation–temperature” coupling and the “wind–humidity” interaction.

**Figure 5:**
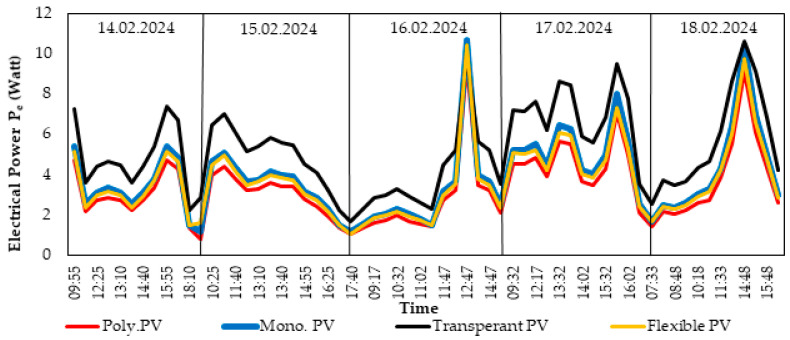
Electrical power (*P_e_*) values produced by PV panels under the climatic conditions of Horseshoe Island, Antarctica (14–18 February 2024).

Figure 5 shows how the electrical power produced by the four different PV panels used during the experiment changed over time. This analysis reveals how the different panel types perform in terms of energy production potential under the challenging Antarctic conditions. According to the data, the MonoPV panel generated the highest electrical power. The maximum power output value of 10.69 W belongs to MonoPV, indicating that this panel has a more efficient electrical conversion capacity compared to the other types. The PolyPV, FlexiblePV, and TransparentPV panels followed, with average power production values of 6.3 W, 8.11 W, and 5.9 W, respectively. The average power production of the TransparentPV panel was lower compared to the others; this suggests that the panel’s light transmissibility feature could be a factor reducing its efficiency. On the other hand, the performance of the FlexiblePV panel, with an average power production similar to the other panels, shows that its flexible structure does not significantly hinder its energy production capacity.

Figure 3 and Figure 4 present environmental factors (solar radiation, relative humidity, temperature, and wind speed) that directly influence the panel performance shown in Figure 5. Changes in solar radiation values are the most significant factor explaining the fluctuations in PV panel power production. In Figure 4, the maximum solar radiation measured was 442 W/m^2^, while environmental variables such as relative humidity indirectly shaped the impact of radiation. These conditions may have contributed to the high efficiency of the MonoPV panel. Similarly, the performance of panels such as PolyPV and FlexiblePV shows their ability to produce consistent power even under more moderate radiation values, highlighting their adaptability.

Wind speed and ambient temperature (Figure 3) are other critical factors affecting the surface temperature of the panels and, consequently, their energy production efficiency. For example, high wind speeds (maximum 5.8 m/s) may have contributed to cooling the panels, preventing overheating. This could have supported the high-efficiency operation of panels like MonoPV.

**Figure 6:**
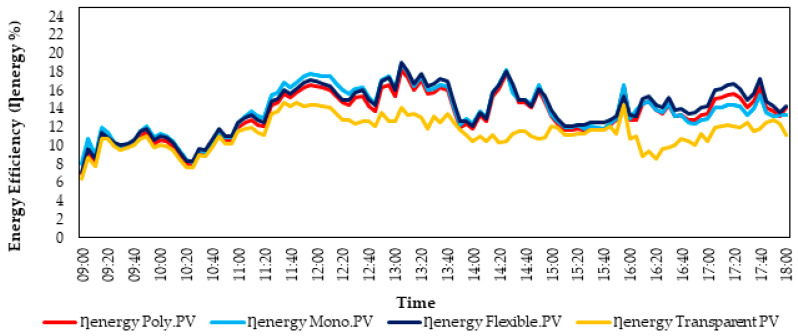
Changes in Energy Efficiency Values of PV Solar Panels During the Experiment.

The changes in the energy efficiency values of PV panels over time are presented in Figure 6. According to Figure 6, the average energy efficiency values of the PolyPV, MonoPV, FlexibleMonoPV, and TransparentPV panels are 13.3%, 13.7%, 13.8%, and 11.4%, respectively. Although the MonoPV panel produced the highest electrical power, the FlexibleMonoPV panel exhibited the highest efficiency value at 19.67%.

### Multi-Objective Optimization-Based Random Rule Search and Modified SPEA-2 Algorithm Results

The multi-objective random rule search-based method used in this study is a supervised data mining technique that includes training and testing processes. The existing dataset represents the search space in this method, and the random search conducted during the training phase results in the generation of the Pareto curve. The candidates forming this curve are the proposed solutions that will be presented to the decision maker. These solutions, which have trade-offs based on data mining metrics, also have their own associated rules. Subsequently, these rules are tested on the test data. All evaluations conducted during the training phase are repeated in the test phase, and the scope of the rules is examined. Naturally, there will be no Pareto curve in the test process, so the scores from the training phase are taken into account.

In the experiments, the dataset was divided into 70% for training and 30% for testing. The classes used in the dataset are HIGH (H), MEDIUM (M), and LOW (L). For these class metrics, the electrical power production values of the PV panels are considered. For HIGH (H), values between 25 W and 15 W, for MEDIUM (M), values between 15 W and 10 W, and for LOW (L), values below 10 W are taken into account. Five independent experiments were conducted for each dataset, and statistical results are provided. Additionally, to enable comparisons, the results of 9 different machine learning algorithms are also presented. The machine learning algorithms used are Naive Bayes (NB), Decision Tree (DT), Support Vector Machine (SVM), k-Nearest Neighbor (kNN), Stacking, Vote, JRip, OneR, and ZeroR.

The machine learning models for comparison were selected to represent established and complementary families in the literature, in order to objectively test the proposed interpretable multi-purpose rule mining approach against different inductive bias and complexity classes: probabilistic (Naive Bayes), tree-based (Decision Tree), margin-based kernel (SVM), example-based (k-NN), rule-based (JRip, OneR), ensemble/meta-learners (Stacking, Voting), and class-frequency-based baseline (ZeroR). This combination provides a broad benchmarking ground that covers linear and nonlinear relationships, bias-variance trade-offs, and the interpretability spectrum, enabling a balanced assessment in terms of robustness, generalizability, and explainability, even under data- or resource-constrained conditions.

Other parameters used in the experiments are listed in Table 5. Table 5 outlines the parameters used in independent experiments. It is noted that each experiment for each dataset was carried out with five independent repetitions. The training and test dataset split ratio was set to 70–30, which indicates that enough data was allocated to evaluate the overall learning performance of the model. The number of individuals (N) used in the experiments was set to 20, which shows a balanced approach to model diversity and the overall optimization process. The crossover probability was set to 0.8, and the mutation probability was set to 0.1, reflecting a preference for high crossover and low mutation rates in genetic algorithm-based optimization processes. The maximum number of iterations was set to 300, indicating that sufficient repetitions were conducted for the model to reach an optimal solution. Additionally, the λ and γ parameters were set to 0.5, which ensures balanced weighting factors during the multi-objective optimization process. Other parameters (***φ*** and **∂**) were defined within specific ranges, demonstrating that a wide parameter space was evaluated during the optimization process.

The settings in Table 5 were selected based on the common “high crossover/low mutation” preference in the literature for genetic search and the necessary exploration–exploitation balance required in our multi-objective (Pareto-front) rule mining framework [29]. The training/test ratio is 70%/30% (the “30–70%” string error in the table header has been corrected), and five independent experiments were run for each dataset. In the SPEA-2 search, the population/archive was set to 20/20, the crossover and mutation probabilities to 0.8/0.1, the maximum iterations to 300, and the threshold/weight parameters to λ = γ = 0.5; these values are appropriate for established applications in GA-based improvements and were determined to ensure convergence [29,30]. Additionally, other parameters, such as φ and, ∂ were scanned within specific ranges to cover a broad search space. As a methodological background, the posteriori approach and adaptation of SPEA-2 to rule mining were preferred; this ensures that the selected settings work consistently with multi-objective optimization.

Overall, the parameters presented in Table 5 indicate that the independent experiments were carefully designed and conducted in a controlled and stable manner to obtain reliable results. When presenting the experimental results, the training phase results will be discussed first, along with the Pareto curves obtained from the independent experiments and the sample rules derived from them. Then, the training and test scores of these rules will be provided, followed by a statistical summary of all independent experiments. Finally, the classical machine learning algorithm results for each dataset will be shared.

**Figure 7:**
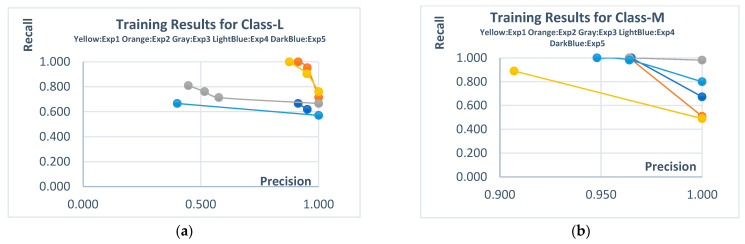
Pareto solutions obtained from the training stages of independent experiments (Transparent PV data set). (**a**) **Class-L**; (**b**) **Class-M**.

The initial experiments were conducted on the Transparent PV panel dataset for each class. For this dataset, which includes only the L and M classes, the Pareto solutions obtained from independent experiments and their corresponding automatically extracted sample rules are presented in Figure 7 and Table 6. The obtained Pareto solutions clearly demonstrate the trade-offs between Recall (Rec) and Precision (Pre). For the L class, candidate solutions with high scores in both Rec and Pre stand out. Although there are also high-scoring candidate solutions for the M class, their precision values remained relatively higher compared to recall. The rules derived for this dataset maintained their performance during the test phase, as they did in the training phase. This indicates the consistency and robustness of the generated rules.

Table 6 presents automatically generated rules for transparent PV panels based on commonly used environmental and operational parameters in PV systems. These rules are defined using variables such as temperature (TEMP), solar radiation (UV), humidity (HUM), wind speed (SPEED), and panel surface temperature (TF), all of which are extensively studied in the literature for their effects on PV power generation. Temperature is a critical factor influencing the performance of PV cells, as increased temperatures reduce the open-circuit voltage and consequently lower the power output [30]. UV is the primary driver of electrical power generation and is directly associated with increased efficiency [31]. Wind speed can contribute positively by cooling the panel surface and mitigating temperature-induced efficiency losses [32]. On the other hand, high humidity can lead to condensation or dirt accumulation on the panel surface, negatively affecting power generation [33]. The presented rules offer a valuable decision-support mechanism by identifying the environmental conditions that influence the performance of transparent PV panels through the combination of these factors.

When examining the coefficients of the variables included in the rules, it is observed that the TEMP and TF parameters have high absolute values, indicating that panel surface temperature has the strongest influence on power generation. The UV parameter generally has a positive effect, showing that UV increases power output, which aligns with the well-established understanding that radiation levels are one of the most critical factors in determining energy production in PV systems. The fact that wind speed (SPEED) shows small but positive coefficients in some models suggests that the cooling effect of wind on the panel is considered by the model, although its overall impact appears to be limited. These results are consistent with findings from various experimental studies on different PV systems in the literature, demonstrating that the rules derived for transparent PV panels successfully reflect the influence of environmental parameters. The references provided in the literature support the importance of the parameters listed in Table 6 for PV systems.

The statistical summary of all independent experiments conducted on this dataset is presented in Table 7, while the results of other machine learning methods are shown in Table 8. In the experiments, it was observed that the Precision and Recall values for Class-M were high, and this performance was maintained in the test data as well. Rule consistency was preserved in both phases.

When compared with other machine learning methods, the best results for Class-L were achieved by SVM and kNN. For Class-M, the proposed method and kNN stood out with the highest scores. The remaining methods either produced non-dominated solutions or were outperformed. A key point to note here is that none of the classical machine learning methods were able to provide alternative solutions that could explain (interpret) their results to the decision-maker as clearly as the proposed method. In other words, the proposed approach not only achieved highly competitive scores but also succeeded in delivering more interpretable and trade-off-inclusive solutions.

**Figure 8:**
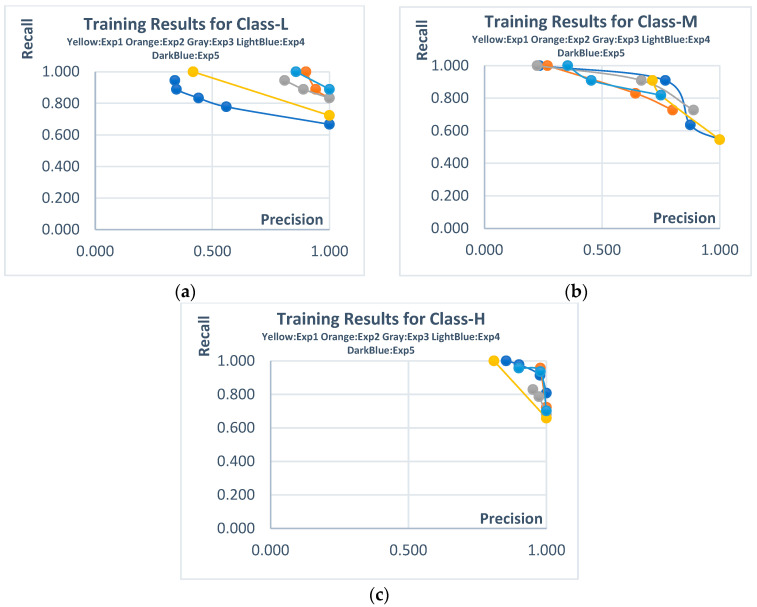
Pareto solutions obtained from the training stages of independent experiments (Mono PV dataset). (**a**) **Class-L**; (**b**) **Class-M**; (**c**) **Class-H**.

The next experiments were conducted using the Mono PV dataset, which includes the L, M, and H classes. The Pareto solutions and example rules obtained for the training phase of these experiments are presented in Figure 8 and Table 9. For Class-L and Class-H, both precision and recall values remained at relatively high levels, with trade-offs forming within this range. Although for Class-M, trade-offs above 0.7 were achieved for both metrics, candidate solutions with lower metric values were also included on the Pareto front.

When examining the automatically generated rules for all three classes in Table 9, it is evident that the panel surface temperature (TM) has a high absolute coefficient value, indicating the dominant effect of temperature on PV panel performance. The literature notes that high temperatures reduce PV cell performance because increasing temperature lowers the bandgap of the semiconductor material, resulting in a lower open-circuit voltage (Voc) [34]. Additionally, UV is widely recognized as the most impactful variable on PV energy production, with higher radiation levels directly increasing the short-circuit current (Isc) and contributing to higher electrical power output [35]. In this table, the UV variable also has a positive coefficient, confirming its critical role in power generation, consistent with the literature.

On the other hand, SPEED may have a cooling effect in some cases, aiding in reducing the panel’s surface temperature and potentially enhancing efficiency [36]; however, this effect is generally observed at low levels. Upon reviewing the coefficients in Table 9, it is clear that temperature (TM) and UV are the most influential factors in PV power output, highlighting the sensitivity of PV panels to thermal and radiative environmental conditions. Although the absolute coefficient value of wind speed is relatively low, the literature suggests it can indirectly support power production by cooling the panel surface [37]. The humidity (HUM) variable tends to have a negative effect due to reduced light transmittance and potential surface contamination, and this study also observes it as a limiting factor with a low but negative coefficient.

Overall, the findings in Table 9 are consistent with various PV performance studies in the literature, indicating that monocrystalline PV panels are particularly sensitive to temperature and UV, and that optimizing environmental variables plays a critical role in enhancing panel efficiency.

The statistical results of all independent experiments conducted on the Mono PV dataset are presented in Table 10, while the outcomes of other machine learning (ML) algorithms are shown in Table 11. The proposed method successfully generated non-dominated candidate solutions across all classes when compared to other methods. It was observed that some classical ML algorithms, particularly for Class-L and Class-M, failed to perform classification. Although the kNN algorithm stood out compared to other classical methods in this dataset, it was still unable to dominate the proposed method. As is well known, kNN is a black-box model, and it lacks interpretability, which limits its practical usefulness.

**Figure 9:**
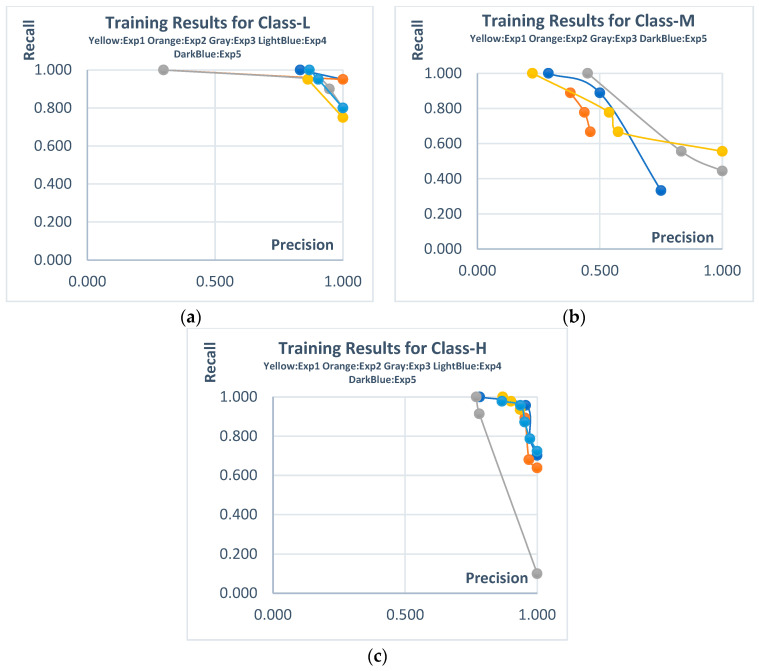
Pareto solutions obtained from training stages of independent experiments (Poly PV dataset). (**a**) **Class-L**; (**b**) **Class-M**; (**c**) **Class-H**.

The Pareto solutions obtained from the training phases of the experiments conducted on the Poly PV dataset and their corresponding automatically generated rules are presented in Figure 9 and Table 12, respectively. This dataset also includes data belonging to the L, M, and H classes. When examining the Pareto solutions, it is observed that high-scoring trade-offs were achieved for both metrics in Class-L and Class-H. However, the widest Pareto solution space was formed for Class-M. When looking at the rule scores produced by the Pareto solutions for the respective classes, the performance achieved during the training phase was generally preserved in the test phase for Class-M and Class-H, while it differed for Class-L.

Based on the explanations provided by the automatically generated rules for decision-makers, Table 12 presents the statistical summary results of independent experiments for polycrystalline (Poly) PV panels, along with a detailed analysis of the effects of environmental variables on electrical power generation. In particular, the impacts of factors such as TEMP, UV, panel surface temperature (TT), SPEED, and HUM are evaluated.

It is widely acknowledged in the literature that UV is directly related to PV panel power generation [38]. However, studies have shown that increasing temperature reduces the efficiency of polycrystalline panels [39], as higher temperatures reduce the bandgap of the semiconductor material, thereby lowering the open-circuit voltage (Voc). Similarly, in this table, the high absolute coefficient of panel surface temperature (TT) confirms the influence of temperature on the energy production of polycrystalline PV panels. The UV variable generally has a positive coefficient, indicating the system’s sensitivity to UV in power production. On the other hand, the effect of SPEED has been evaluated positively in some studies, as airflow can reduce panel surface temperature and thus offset the negative effects of heat [40].

When the coefficients of the parameters are examined in detail, it is observed that UV has the most significant positive effect on polycrystalline PV panels, supporting the conclusion that radiation levels are one of the most critical factors determining energy production in PV systems. Panel surface temperature (TT) has the highest negative absolute value, indicating that temperature is a factor that reduces panel efficiency. The SPEED variable, although it somewhat reduces the negative effect created by temperature on the panel surface, has been found to have a relatively low overall impact. The humidity (HUM) factor, as noted in the literature, generally causes pollution or condensation on the panel surface, reducing light transmissibility and thus power generation [41]. In this context, the findings presented in Table 12 largely align with studies conducted in the literature, revealing that polycrystalline PV panels are highly sensitive to temperature fluctuations, highly responsive to UV, and that wind speed can have a positive contribution in specific conditions.

The statistical summary data for all independent experiments conducted on this dataset, along with the results for other ML methods, are provided in Table 13 and Table 14, respectively. The consistency of Class-M and Class-H in both the training and test phases is evident from the average values of the independent experiment results. However, a significant drop in recall scores for Class-L can be observed here. When examining the results of other machine learning methods, it is seen that the proposed method produced highly competitive results with both dominated and non-dominated solutions in Class-M and Class-H. However, for Class-L, some classic ML methods (such as Naive Bayes, SVM, and kNN) achieved better results.

**Figure 10:**
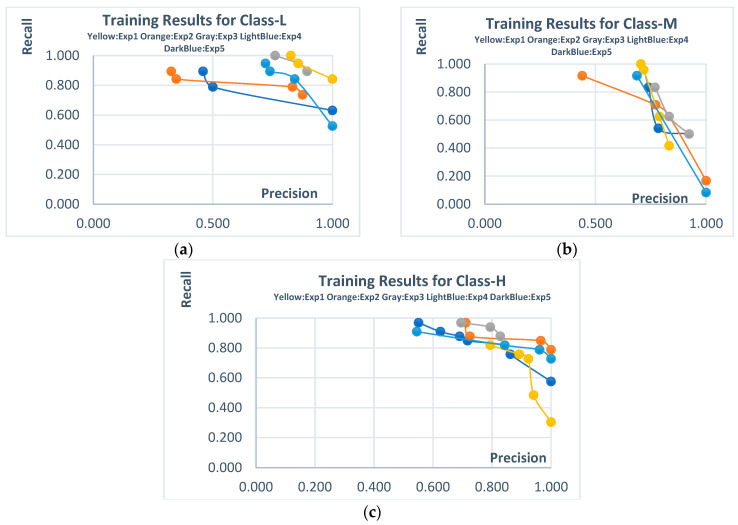
Pareto solutions obtained from the training stages of independent experiments (Flexible PV data set). (**a**) **Class-L**; (**b**) **Class-M**; (**c**) **Class-H**.

The latest experiments have been conducted again using flexible mono (Flexible) PV data containing three classes. The outputs from the training phase and sample rules for this data are presented in Figure 10 and Table 15. The Pareto distributions have remained within a wide range for both metrics, and solutions with different trade-offs have been presented to the decision-maker. The performance of the rules in both phases has been generally maintained across all classes. When the rules generated by the Pareto solutions presented in Table 15 are examined, it is seen that the electrical power production value of the flexible PV panel depends on variables such as TEMP, UV, HUM, SPEED, and panel surface temperature (TS). The effects of these parameters on PV systems and environmental factors are frequently discussed in the literature, and they are known to be among the most critical variables for power generation in PV panels [42]. In particular, the determining role of UV in electrical power production has been emphasized in many studies, and it has been shown that higher radiation levels directly increase power output [43]. When the rules in Table 15 are examined, it can be observed that the rules based on the range of UV have high accuracy and recall values. For example, rules within the range of “145.377 < UV < 422.781” show that power production significantly decreases when radiation drops below a certain threshold. Similarly, it has been observed that rules that keep the surface temperature (TS) within a certain range yield high performance. These findings align with recent studies showing that the surface temperature of the PV panel is a determining factor for power production [44].

The data in Table 15 reveal the explainability levels and success rates of the rules for different classes (L, M, H). Particularly, it has been observed that wind speed (SPEED) is a critical variable for classification within certain ranges. An increase in wind speed at specific levels can reduce the PV panel temperature, thereby providing stability in power generation [45]. It is seen that the rules exhibit similar performances on both training and test data, suggesting the model has strong generalization capability. Specifically, rules within the ranges “1.214 < TEMP < 4.409” and “53.800 < HUM < 75.469” have shown high success for the M class, indicating that balancing temperature and humidity helps maintain consistency in power production. Overall, the rules in Table 15 successfully capture the effects of environmental variables on power generation and produce results consistent with approaches proposed in the literature [46].

The statistical summary table in Table 16 proves that the rule scores produced in all classes maintain their stability. When compared with the other ML method results (Table 17), it is seen that the proposed method provides explainability as in other data sets and produces competitive solutions with high scores. The method that produces non-dominated solutions compared to the results of other methods in all classes. It generally competed with kNN. In all experiments, the most prominent method among the classical methods was generally kNN.

## 4. Conclusions

This study provides a comprehensive assessment by combining a controlled field comparison of four PV module types (monocrystalline, polycrystalline, flexible mono, semi-transparent) using simultaneous PV-meteorological time series on Antarctica/Horseshoe Island with interpretable multi-purpose modeling. Electrical quantities (V–I–P) along with solar radiation, air temperature, wind speed, and relative humidity were measured in the field; analyses were limited to daytime periods only. The conclusions are summarized below.

***Experimental findings:*** Power outputs showed significant intraday and meteorology-driven variability; the overall ranking by panel type was observed as MonoPV > FlexiblePV ≈ PolyPV > TransparentPV. No H class formed in the semi-transparent subgroup; class distributions were reported specific to panel types. Panel-based energy performance was summarized using average Pmax and energy efficiency indicators.

***Modeling and optimization:*** Modified SPEA-2 was shown to jointly optimize precision and recall on the Pareto-front of the rules; a distinct equilibrium (knee-point) solution was identified on the front. In a sample test slice, precision = 0.973 and recall = 0.903 were obtained for semi-transparent/Class-M.

***Comparative performance:*** The proposed rule-based approach produced competitive results against standard baselines, such as kNN and SVM. In addition to precision–recall metrics, F1, accuracy, balanced accuracy, MCC, Cohen’s κ, specificity (TNR), and NPV were also reported to evaluate class imbalance and robustness comprehensively.

***Explainability and applicability:*** The learned rules provided physical intuition consistent with radiation/temperature/wind regimes; they produced actionable thresholds that could directly inform threshold-based timing, curtailment, and mode switching decisions during rapid weather changes.

***Application and future work:*** The findings provide a replicable dataset and an interpretable methodological framework for the design and operation of PV systems in polar/cold climates. The study of plans involves assessing applications in real-time through a 25 W-referenced normalized cost table (TL/W), utilizing polar winter data, and integrating PV-wind hybrids.

These results demonstrate that flexible monocrystalline PV panels are the most suitable renewable energy solution for temporary or seasonal research stations in Antarctica. Additionally, the use of explainable artificial intelligence methods has brought transparency to energy decision-making processes, offering a sustainable and scientifically grounded roadmap for reliable energy production in extreme environmental conditions. The data obtained from this study serve as a valuable reference for evaluating the applicability of renewable energy systems in harsh environmental conditions in future research. Further studies could focus on extending data collection periods and integrating additional environmental factors into models to improve performance predictions. This research provides a solid foundation for developing strategies to optimize the use of renewable energy systems in extreme regions like Antarctica.

## Figures and Tables

**Table 1:** Specifications of the measurement equipment used in the experiments.

Parameter	Measurement Device	Model	Accuracy
Cell Temperature	Laser Sensor temperature (measurement range: −20 °C~450 °C, working temperature: −40 °C~85 °C)	DFRobot	±0.1 °C
Temperature and Humidity	Transmitter Sensor (measurement range: 0–100%, working temperature: −40 °C~70 °C)	Eliwell-EWHS 2840	±5%
Wind velocity	Transmitter Sensor (measurement range: 0–75 m/s çalışma sıcaklığı: −40 °C~100 °C)	Dwyer	±4%
Solar radiation	Solar radiation (measurement range: 0–1500 W/m^2^, working temperature: −50 °C~85 °C)	HUKSEFLUX	±1 W/m^2^
Electric Power	Power analysis sensor (0–1000 W, 0–100 V, 0–10 A)	KYORITSU 8309	±1%

**Table 2:** Technical details of PV panel.

Electrical Characteristics	Mono PV	Poly PV	Transparent PV	Flexible PV
Power	25 W	25 W	25 W	25 W
Cell number	36	36	20	20
Open-Circuit voltage (Voc)	24.84 V	23.32 V	14.21 V	14.21 V
Maximum Voltage (Vmp)	20.70 V	18.60 V	12.40 V	12.40 V
Short-Circuit Current (Isc)	1.27 A	1.38 A	2.24 A	2.24 A
Maximum Current (Imp)	1.21 A	1.22 A	2.06 A	2.06 A
Maximum System Voltage	1000 V	1000 V	1000 V	1000 V

**Table 3:** Calculated Uncertainty Values for PV Panels.

Uncertainty	ɳ_energy_	Electrical Power
MPV	5.4%	1.13%
PPV	5.2%	1.14%
FPV	5.5%	1.11%
TPV	5.8%	1.12%

**Table 4:** Calculation of parameters used for Precision and Recall values.

Left Side of the Rule	Right Side of the Rule	Update Process
Left side of the rule Correct	Right side of the rule Correct	TP = TP + 1
Left side of the rule Incorrect	Right side of the rule Incorrect	TN = TN + 1
Left side of the rule Correct	Right side of the rule Incorrect	FP = FP + 1
Left side of the rule Incorrect	Right side of the rule Correct	FN = FN + 1

**Table 5:** Experimental parameters.

Parameters	Value
**Number of independent experiments for each dataset**	5
**Splitting ratio for Training-Test datasets**	70–30%
N **and** $\bar{N}$	20/20
λ **and** γ	0.5
φ	5
**∂**	$\left[0,\;2\right]\in R$
**Crossover and mutation probability**	0.8 and 0.1
**Maximum iteration**	300

**Table 6:** Some automatically generated rules for transparent PV panel electrical power generation set.

Training		Test		Rule
Pre	Rec	Pre	Rec	
0.636	0.666	0.666	0.666	if 0.645 < TEMP < 5.400 and 2.606 < TE < 8.767 then L
0.913	1.000	**0.900**	**1.000**	if 99.000 < UV < 221.388 then L
**0.950**	**0.904**	0.857	0.666	if 0.131 < TEMP < 3.369 and 0.818 < SPEED < 2.321 then L
0.875	1.000	**1.000**	**1.000**	if 0.100 < TEMP < 3.046 then L
**0.965**	**1.000**	0.958	0.958	if 2.042 < TEMP < 5.700 then M
**1.000**	**0.800**	**1.000**	**0.750**	if 3.631 < TEMP < 5.128 and 2.634 < TE < 15.890 then M
0.964	0.981	0.960	1.000	if 310.404 < UV < 372.092 then M

**Table 7:** Performance metrics statistics for independent experiments of Transparent PV dataset.

	Class-L				Class-M			
	Training		Test		Training		Test	
	Pre	Rec	Pre	Rec	Pre	Rec	Pre	Rec
Min	0.636	0.666	0.666	0.666	0.964	0.800	0.958	0.750
Max	0.950	1.000	1.000	1.000	1.000	1.000	1.000	1.000
Avg	0.844	0.893	0.856	0.833	0.976	0.927	0.973	0.903
Mean	0.894	0.952	0.879	0.833	0.976	0.927	0.973	0.903
Std	0.142	0.158	0.140	0.193	0.021	0.110	0.024	0.134

**Table 8:** Results from classical machine learning (ML) methods for the Transparent PV dataset.

Model	Class	Precision	Recall
Naive Bayes	L	0.867	1
	M	**1**	**0.9**
SVM	L	**1**	**0.923**
	M	0.952	1
kNN	L	**1**	**1**
	M	**1**	**1**
Stacking	L	-	0
	M	0.606	1
VOTE	L	-	0
	M	0.606	1
JRip	L	**0.929**	**1**
	M	1	0.95
OneR	L	0.929	1
	M	1	0.95
ZeroR	L	-	0
	M	0.606	1
DT	L	0.929	1
	M	**1**	**0.95**

**Table 9:** Some automatically generated rules for mono PV panel electrical power generation set.

Training		Test		Rules
Pre	Rec	Pre	Rec	
1.000	0.680	0.928	0.650	if 1.868 < TEMP < 4.906 and 10.081 < TM < 13.653 then H
**0.810**	**1.000**	**0.730**	**0.950**	if 1.668 < TEMP < 5.700 and 345.829 < UV < 422.1 then H
0.900	0.957	0.850	0.850	if 3.541 < TEMP < 5.631 then H
0.800	0.727	0.600	0.600	if 0.972 < SPEED < 2.746 and 4.191 < TM < 9.086 then M
0.889	0.727	0.500	0.400	if 274.955 < UV < 400.706 and 5.778 < TM < 14.519 then M
1.000	0.667	1.000	0.375	if 74.888 < HUM < 78.989 and 124.612 < UV < 324.316 then L
**1.000**	**0.834**	**1.000**	**0.750**	if 0.277 < TEMP < 3.099 and 108.197 < UV < 407.400 then L
1.000	0.723	1.000	0.750	if 0.117 < TEMP < 4.430 and 127.116 < UV < 371.684 then L

**Table 10:** Performance metrics statistics for mono PV dataset independent experiments.

	Class-L				Class-M				Class-H			
	Training		Test		Training		Test		Training			Test
	Pre	Rec	Pre	Rec	Pre	Rec	Pre	Rec	Pre	Rec	Pre	Rec
Min	0.340	0.667	0.421	0.375	0.224	0.081	0.267	0.200	0.810	0.659	0.083	0.650
Max	1.000	1.000	1.000	1.000	1.000	1.000	0.667	1.000	1.000	1.000	1.000	0.950
Avg	0.767	0.874	0.795	0.767	0.649	0.783	0.447	0.600	0.952	0.852	0.829	0.811
Mean	0.889	0.889	0.857	0.750	0.732	0.864	0.432	0.600	0.978	0.872	0.894	0.825
Std	0.264	0.099	0.230	0.176	0.263	0.243	0.144	0.263	0.061	0.126	0.229	0.108

**Table 11:** Results from classical machine learning (ML) methods for the mono PV dataset.

Model	Class	Precision	Recall
Naive Bayes	L	0.75	0.9
	M	0.444	0.571
	H	**1**	**0.75**
SVM	L	0.75	0.9
	M	0.5	0.143
	H	**0.842**	**1**
kNN	L	**0.909**	**1**
	M	0.625	0.714
	H	0.929	0.813
Stacking	L	-	0
	M	-	0
	H	0.485	1
VOTE	L	-	0
	M	-	0
	H	0.485	1
JRip	L	0.769	1
	M	0.667	0.286
	H	0.882	0.938
OneR	L	0.75	0.9
	M	0.6	0.429
	H	0.938	0.938
ZeroR	L	-	0
	M	-	0
	H	0.485	1
DT	L	0.714	1
	M	0.5	0.143
	H	0.882	0.938

**Table 12:** Some automatically generated rules for Poly PV panel electrical power generation set.

Training		Test		Rules
Pre	Rec	Pre	Rec	
**1.000**	**0.950**	0.800	0.444	if 72.322 < HUM < 79.100 and 156.000 < UV < 365.647 and 0.367 < SPEED < 2.841 then L
0.904	0.950	0.667	0.444	if 188.710 < UV < 309.444 then L
0.750	0.333	1.000	0.500	if 69.441 < HUM < 76.100 and 580.680 < UV < 327.409 and 2.412 < TP < 12.205 then M
1.000	0.444	**1.000**	**0.750**	if 381.656 < UV < 328.541 and 1.057 < SPEED < 2.692 and 6.536 < TP < 14.488 then M
0.538	0.778	0.600	0.750	if 2.061 < TEMP < 3.907 and 66.240 < HUM < 78.309 and 323.894 < UV < 376.287 and 3.934 < TP < 10.359 then M
1.000	0.556	1.000	0.750	if 3.040 < TEMP < 4.228 and 66.984 < HUM < 78.563 and 290.078 < UV < 358.190 and 4.490 < TP < 10.427 then M
0.957	0.957	**1.000**	**1.000**	if 328.623 < UV < 406.308 then H
0.969	0.680	1.000	0.450	if 4.163 < TEMP < 4.829 then H
**0.936**	**0.936**	**1.000**	**0.950**	if 54.100 < HUM < 68.320 then H
1.000	0.085	1.000	0.050	if 2.206 < SPEED < 2.782 and 7.376 < TP < 9.811 then H

**Table 13:** Performance metrics statistics for independent experiments of the Poly PV dataset.

	Class-L				Class-M				Class-H			
	Training		Test		Training		Test		Training		Test	
	Pre	Rec	Pre	Rec	Pre	Rec	Pre	Rec	Pre	Rec	Pre	Rec
Min	0.298	0.750	0.290	0.222	0.225	0.100	0.250	0.500	0.770	0.085	0.791	0.050
Max	1.000	1.000	1.000	1.000	1.000	1.000	1.000	1.000	1.000	1.000	1.000	1.000
Avg	0.840	0.923	0.677	0.487	0.612	0.659	0.648	0.783	0.921	0.848	0.943	0.745
Mean	0.904	0.950	0.750	0.444	0.538	0.667	0.600	0.750	0.937	0.936	1.000	0.950
Std	0.248	0.086	0.199	0.255	0.261	0.255	0.318	0.129	0.073	0.211	0.077	0.273

**Table 14:** Results from classical machine learning (ML) methods for the Poly PV dataset.

Model	Class	Precision	Recall
Naive Bayes	L	**1.000**	**0.923**
	M	0.444	1.000
	H	1.000	0.750
SVM	L	**1.000**	**0.923**
	M	0.500	0.250
	H	0.842	1.000
kNN	L	1.000	0.846
	M	0.375	0.750
	H	0.929	0.813
Stacking	L	-	0.000
	M	-	0.000
	H	0.485	1.000
VOTE	L	-	0.000
	M	-	0.000
	H	0.485	1.000
JRip	L	1.000	0.923
	M	0.500	0.500
	H	0.882	0.938
OneR	L	1.000	0.923
	M	0.500	0.750
	H	0.933	0.875
ZeroR	L	-	0.000
	M	-	0.000
	H	0.485	1.000
DT	L	**0.929**	**1.000**
	M	0.500	0.250
	H	0.882	0.938

**Table 15:** Pareto solutions obtained from the training stages of independent experiments (Flexible PV data set).

Training		Test		Rules
Pre	Rec	Pre	Rec	
0.459	0.894	0.368	1.000	if 145.377 < UV < 179.781 then L
**0.910**	**0.842**	**0.857**	**0.857**	if 72.213 < HUM < 80.143 then L
1.000	0.167	0.750	0.272	if 3.162 < TEMP < 5.012 and 56.104 < HUM < 72.513 and 281.576 < UV < 315.865 and 1.195 < SPEED < 2.594 then M
0.789	0.625	0.750	0.545	if 1.333 < TEMP < 4.394 and 280.028 < UV < 304.198 and 1.099 < SPEED < 3.559 and 2.985 < TS < 10.092 then M
0.833	0.417	**1.000**	**0.272**	if 273.817 < UV < 354.004 and 2.194 < SPEED < 3.323 and 3.999 < TS < 11.658 then O
0.688	0.916	0.615	0.727	if 1.214 < TEMP < 4.409 and 53.800 < HUM < 75.469 and 1.159 < SPEED < 3.800 then M
0.690	0.878	0.619	0.866	if 55.134 < HUM < 69.109 and 0.233 < SPEED < 3.094 then H
**1.000**	**0.575**	**1.000**	**0.533**	if 11.969 < TS < 14.016 then H
0.794	0.818	0.769	0.667	if 382.313 < UV < 418.588 then H
1.000	0.303	0.750	0.200	if 3.905 < TEMP < 5.561 and 0.820 < SPEED < 1.470 then H

**Table 16:** Performance metrics statistics of flexible PV data set for independent experiments.

	Class-L				Class-M				Class-H			
	Training		Test		Training		Test		Training		Test	
	Pre	Rec	Pre	Rec	Pre	Rec	Pre	Rec	Pre	Rec	Pre	Rec
Min	0.326	0.526	0.291	0.285	0.428	0.083	0.010	0.010	0.545	0.303	0.500	0.133
Max	1.000	1.000	1.000	1.000	1.000	1.000	1.000	1.000	1.000	0.969	1.000	1.000
Avg	0.755	0.848	0.802	0.823	0.767	0.665	0.727	0.689	0.822	0.798	0.779	0.774
Mean	0.833	0.894	0.875	0.857	0.769	0.625	0.714	0.727	0.843	0.818	0.769	0.866
Std	0.218	0.126	0.262	0.223	0.151	0.275	0.265	0.278	0.144	0.160	0.152	0.251

**Table 17:** Results obtained from classical machine learning (ML) methods for flexible PV data sets.

Model	Class	Precision	Recall
Naive Bayes	L	**0.714**	**1.000**
	M	0.600	0.600
	H	**1.000**	**0.692**
SVM	L	0.750	0.900
	M	0.583	0.700
	H	**1.000**	**0.692**
kNN	L	**0.909**	**1.000**
	M	0.889	0.800
	H	0.923	0.923
Stacking	L	-	0.000
	M	-	0.000
	H	0.394	1.000
VOTE	L	-	0.000
	M	-	0.000
	H	0.394	1.000
JRip	L	**0.714**	**1.000**
	M	0.600	0.600
	H	1.000	0.692
OneR	L	**0.769**	**1.000**
	M	0.636	0.700
	H	1.000	0.692
ZeroR	L	-	0.000
	M	-	0.000
	H	0.394	1.000
DT	L	0.714	1.000
	M	0.600	0.600
	H	**1.000**	**0.692**

## Data Availability

The raw data supporting the conclusions of this article will be made available by the authors upon request.
